# Expression of dihydropyrimidine dehydrogenase (DPD) and hENT1 predicts survival in pancreatic cancer

**DOI:** 10.1038/s41416-018-0004-2

**Published:** 2018-03-08

**Authors:** N. O. Elander, K. Aughton, P. Ghaneh, J. P. Neoptolemos, D. H. Palmer, T. F. Cox, F. Campbell, E. Costello, C. M. Halloran, J. R. Mackey, A. G Scarfe, J. W. Valle, A. C. McDonald, R. Carter, N. C. Tebbutt, D. Goldstein, J. Shannon, C. Dervenis, B. Glimelius, M. Deakin, R. M. Charnley, Alan Anthoney, M. M. Lerch, J. Mayerle, A. Oláh, M. W. Büchler, W. Greenhalf, for the European Study Group for Pancreatic Cancer

**Affiliations:** 1 From the Cancer Research U.K. Liverpool Cancer Trials Unit, University of Liverpool, Liverpool, UK; 2 The Department of Surgery, University of Heidelberg, Heidelberg, Germany; 3 Cross Cancer Institute and University of Alberta, Alberta, Canada; 4 University of Manchester/The Christie NHS Foundation Trust, Manchester, UK; 5 The Beatson West of Scotland Cancer Centre, Glasgow, Scotland UK; 6 Glasgow Royal Infirmary, Glasgow, Scotland UK; 7 Austin Health, Melbourne, Australia; 8 Prince of Wales hospital and Clinical School University of New South Wales, New South Wales, Australia; 9 Nepean Cancer Centre and University of Sydney, Sydney, Australia; 10 The Agia Olga Hospital, Athens, Greece; 11 Department of Immunology, Genetics and Pathology, Uppsala University, Uppsala, Sweden; 12 University Hospital, North Staffordshire, UK; 13 Freeman Hospital, Newcastle upon Tyne, UK; 14 St James’s University Hospital, Leeds, UK; 15 Department of Medicine A, University Medicine Greifswald, Greifswald, Germany; 16 Department of Medicine II, University Hospital of the Ludwig-Maximilians-University, Munich, Germany; 17 The Petz Aladar Hospital, Gyor, Hungary

**Keywords:** Cancer, Tumour biomarkers

## Abstract

**Background:**

Dihydropyrimidine dehydrogenase (DPD) tumour expression may provide added value to human equilibrative nucleoside transporter-1 (hENT1) tumour expression in predicting survival following pyrimidine-based adjuvant chemotherapy.

**Methods:**

DPD and hENT1 immunohistochemistry and scoring was completed on tumour cores from 238 patients with pancreatic cancer in the ESPAC-3(v2) trial, randomised to either postoperative gemcitabine or 5-fluorouracil/folinic acid (5FU/FA).

**Results:**

DPD tumour expression was associated with reduced overall survival (hazard ratio, HR = 1.73 [95% confidence interval, CI = 1.21–2.49], *p* = 0.003). This was significant in the 5FU/FA arm (HR = 2.07 [95% CI = 1.22–3.53], *p* = 0.007), but not in the gemcitabine arm (HR = 1.47 [0.91–3.37], *p* = 0.119). High hENT1 tumour expression was associated with increased survival in gemcitabine treated (HR = 0.56 [0.38–0.82], *p* = 0.003) but not in 5FU/FA treated patients (HR = 1.19 [0.80–1.78], *p* = 0.390). In patients with low hENT1 tumour expression, high DPD tumour expression was associated with a worse median [95% CI] survival in the 5FU/FA arm (9.7 [5.3–30.4] vs 29.2 [19.5–41.9] months, *p* = 0.002) but not in the gemcitabine arm (14.0 [9.1–15.7] vs. 18.0 [7.6–15.3] months, *p* = 1.000). The interaction of treatment arm and DPD expression was not significant (*p* = 0.303), but the interaction of treatment arm and hENT1 expression was (*p* = 0.009).

**Conclusion:**

DPD tumour expression was a negative prognostic biomarker. Together with tumour expression of hENT1, DPD tumour expression defined patient subgroups that might benefit from either postoperative 5FU/FA or gemcitabine.

## Introduction

Pancreatic ductal adenocarcinoma is one of the leading causes of cancer-related death worldwide and will shortly overtake breast cancer as the second leading cause of cancer death in the USA, with limited survival following primary treatment.^1–3^ Following multicentre studies by the European Study Group for Pancreatic Cancer (ESPAC) and others, it is now clear that adjuvant chemotherapy with either 5-fluorouracil with folinic acid (5FU/FA), gemcitabine monotherapy, or gemcitabine plus capecitabine (a 5FU prodrug) for 6 months following pancreatic resection increases long-term survival.^4–10^ Adjuvant S-1, an orally active drug containing tegafur (another 5FU prodrug), has also improved survival in patients from Japan.^11^

Although both 5FU/FA and gemcitabine are efficient at the cohort level, specific individuals may benefit more from either gemcitabine or 5FU/FA. There are currently no established tools to select the optimal treatment for the individual patient with pancreatic cancer. The cellular response to pyrimidine-based chemotherapy is dependent on a series of proteins involved in the trans-membrane uptake and metabolism.^12,13^ Our laboratory has previously reported that high protein expression of human equilibrative nucleoside transporter 1 (hENT1) was associated with improved overall survival of patients in the gemcitabine arm of the ESPAC-3(v2) trial, but not in the 5FU/FA arm.^14^ These results indicated that other markers should be sought to help predict 5FU activity.

Dihydropyrimidine dehydrogenase (DPD) is an enzyme encoded by the gene *DPYD* located on chromosome *1p22,*^15^ which catabolizes 5FU into dihydrofluorouracil.^16^ Metabolites of 5FU interfere with cell function by inhibition of DNA synthesis and repair, RNA transcription and DNA methylation.^16^ The main mechanism of 5FU activation is conversion to fluorodeoxyuridine via thymidylate phosphorylase and then conversion to fluorodeoxyuridine monophosphate (FdUMP) by thymidine kinase. FdUMP inhibits thymidylate synthase, which is important for the folate-homocysteine cycle and purine and pyrimidine synthesis. Other key metabolites are fluorouridine triphosphate and fluorodeoxyuridine triphosphate, which are incorporated into RNA and DNA, respectively. The rate-limiting step of 5FU catabolism is the conversion of 5FU to dihydrofluorouracil by DPD, which is then converted to fluoro-β-ureidopropionate and subsequently to fluoro-β-alanine.^16^ Thus, we could hypothesise that low intra-tumoural DPD expression would favour the production of cytotoxic 5FU metabolites and prolong survival. This hypothesis has received some support from small retrospective studies predominantly involving the composite drug S-1.^17–23^ Gimeracil, a component of S-1, is an inhibitor of DPD that maintains a high concentration of 5FU in blood and tumour tissue.^11^

In the present study, the expression of intra-tumoural DPD was analysed in tissue from patients in the ESPAC-3(v2) trial that had been randomised to 6 months of gemcitabine or 5FU/FA following pancreatic resection. Our primary objective was to test the hypothesis that DPD expression status was a specific marker for 5FU-based chemotherapy. Secondary exploratory objectives tested whether DPD expression could add to the predictive value of hENT1 expression in selecting patients for either gemcitabine or 5FU adjuvant therapy.^14^

## Materials and Methods

### Study design

The translational ESPAC-T studies received ethics committee approval for the characterization of tumour markers for chemotherapy from the Liverpool (Adult) Research Ethics Committee (07/H1005/87). Good Clinical Practice Standard Operating Procedures (SOPs) were employed to minimise study biases with a full audit trail. The ESPAC-3 trial randomised 551 patients to 5FU/FA and 537 to gemcitabine (Neoptolemos et al.^7^)]. This was originally analysed on an intention-to-treat basis but, for the ESPAC-T study, patients in the treatment arms were selected for inclusion only if treatment was actually received. All patients provided written informed consent. This study was conducted and reported in accordance with the REMARK criteria.^24,25^

### Tissue microarray (TMA) manufacture

Tissue arrays were manufactured using SOP’s as previously reported.^14^ The arrays contained tumour cores from patients included in the ESPAC-3(v2) trial and randomised to 5FU/FA or gemcitabine, or from patients from the ESPAC-1/ESPAC-3(v1) trial randomised to observation only. Cores were taken from tumour regions identified by an experienced pancreatic pathologist (FC) using haematoxylin and eosin (H&E)-stained sections. Tissue microarrays were prepared with two cores from each block, with four to eight cores arrayed for each patient. Each of the TMA’s had two cores from each of 88 patients. For all arrays, control cores, comprising three cores each of colon, kidney, liver, normal pancreas, and chronic pancreatitis, were arranged in a fence around the test samples. Each core on each TMA was coded and linked separately to trial identifiers.

### Immunohistochemistry

TMA blocks were cut in 3 µm sections and placed on Superfrost Ultra Plus® slides (Thermo Fisher Scientific Inc., Waltham, MA, USA). Deparaffinisation and antigen retrieval were performed with the PT-Link® system and pH 9.0 target retrieval buffer (Dako, Glostrup, Denmark). All buffers and reagents were provided in the EnVision^TM^ kit (Dako): slides were washed in tris-buffered saline with 0.05% Tween-20 (TBS-T) before peroxidase blocking for 10 min. Following TBS-T washes, samples were incubated with rabbit-anti-DPD diluted 1:2000 for 60 min, followed by incubation with secondary horseradish peroxidase-conjugated antibody for 60 min. Following repeated TBS-T washes, slides were covered in fresh diaminosobenzidine (DAB) working solution for 10 min in room temperature. Slides were washed in TBS-T and distilled water, and counterstained in Haematoxylin Gills III and dehydrated via a series of ethanol gradients and xylene before being mounted under cover slips.

### Validation and quality assessment of the primary anti-DPD antibody

The primary antibody (rabbit-anti-DPD, Abcam *Ab 134922*, Abcam, Cambridge, UK) was validated in accordance with ESPAC-T steering committee policy. Western blot and immunocytochemistry on lysates and paraffin-embedded naive as well as anti-DPD siRNA-treated cell lines confirmed that the antibody was specific and sensitive for the presence or absence of human DPD (Supplementary Figures 1–3, Online Data). Positive-staining tissue cores (healthy liver) and negative-staining tissue cores (healthy colon) were used as internal controls. Negative control slides underwent identical staining procedures, but with the primary antibody replaced by antibody dilution buffer only.

### Scoring

The DPD expression in tumour cell compartments of all samples were scored by one experienced pancreas pathologist (FC) and one trained assistant (EG) according to a 0–3 point system (0 = no staining, 1 = weak, 2 = moderate, 3 = strong staining, with representative images viewed in Supplementary Figures 4A–D). FC and EG were both blinded to patient ID and clinical data. In general, the intra-core variability was low, but if staining intensity within the core was not fully consistent, the most commonly observed pattern was scored. This means that if a core contained only one or two cells that were immunopositive, but the predominant pattern was negative (‘0’), then the core in total was scored ‘0′. Any disagreement in scoring of the immunohistochemistry was resolved through discussion and a consensus decision. Each patient was given a single scoring grade equal to the mean of cores, rounded to the nearest integer. Since a score = 3 was found in only three patients in the entire cohort, scores 2–3 were grouped into the high DPD expressing group, and dichotomous comparisons were consequently performed with the low DPD expressing group (scores = 0–1). The previously collected hENT1 scores for the tumours^14^ were added to the data set to investigate a possible relationship with the DPD scores. The DPD and hENT1 scores were not correlated (Pearson correlation = −0.01).

### Statistical considerations

Survival from the date of randomisation was analysed using Kaplan–Meier curves, with differences between groups assessed using the log rank test.^26,27^ Univariable and multivariable analyses, using a backwards elimination method, were carried out using *Cox* proportional hazards.^28^ A 2-sided significance level of *P* < 0.05 was used throughout. If not otherwise stated, 95% confidence intervals (CI) were presented. To adjust for multiple testing in the combined DPD and hENT1 expression subgroups, Bonferroni correction was performed for these analyses. Analyses were carried out using STATA v14 (StataCorp).

## Results

### Immunohistochemical staining and scoring

We stained tissue cores from 303 patients: 272 patients randomised and treated in the chemotherapy arms of the ESPAC-3(2) trial,^7^ and 31 patients randomised to observation in the combined ESPAC-1/ESPAC-3(v1) trials.^4–7^ Cores from 34 patients from the ESPAC-3(v2) chemotherapy arms and eight patients from the observational arms contained insufficient tissue to score, or only severely damaged tissue. Overall cores from 261 (86.14%) patients were scored, including 238 chemotherapy-treated patients, 115 (20.9% originally randomised) given 5FU/FA and 123 (20.9% originally randomised) given gemcitabine plus 23 patients randomised to observation. Demographics, shown in Supplementary Table 1, are similar to those previously reported for the whole trial population.^7,14^ DPD expression tumour scores in relation to clinical and pathological variables are shown in Supplementary Table 2. Representative images of the different scores and their respective frequencies in the entire population are presented in Supplementary Figure 4.

### Cox regression univariate analyses

Cox proportional hazards univariate analyses of survival by clinico-pathologic risk factors, DPD tumour expression (low expression, score = 0–1; high expression, score = 2–3) and hENT1 expression (low/high, cutoff defined by the median H-score) by treatment arm and collectively are shown in Table 1. Significant prognostic factors for the entire chemotherapy-treated population (both gemcitabine and 5FU/FA) were resection margin status, WHO performance status, lymph node status, tumour stage, tumour invasion into nearby organs, and DPD expression. High DPD expression was associated with reduced survival (hazard ratio [HR] 1.73, 95% CI: 1.21–2.49, *p* = 0.003). This difference was significant in the 5FU/FA arm (HR: 2.07, 95% CI: 1.22–3.53, *p* = 0.007), but not in the gemcitabine arm (HR: 1.47, 95% CI: 0.91–2.37, *p* = 0.119). Tumour expression of DPD was not significantly associated with any of the other clinical or pathological factors analysed (Supplementary Table 2). Tumour expression of hENT1 was not prognostic for the whole chemotherapy cohort (HR: 0.84, 95% CI: 0.63–1.12, *p* = 0.230), but was predictive for improved survival with gemcitabine (HR: 0.56, 95% CI: 0.38–0.82, *p* = 0.003) but not for 5FU/FA (HR: 1.19, 95% CI: 0.80–1.78, *p* = 0.390).

**Table 1:** Cox proportional hazards univariate analyses of survival by clinical and pathological risk factors, DPD tumour expression (low, score = 0–1; high, score = 2–3), and hENT1 tumour expression (high vs. low defined by median H-score)

Univariate analysis				
	Hazard ratio (95% Confidence Interval)			
	Chemotherapy			Total
Characteristic		5-fluorouracil /folinic acid	Gemcitabine	
Resection margin		*n* = 115	*n* = 123	*n* = 238
	Negative	1	1	1
	Positive	2.13 (1.41–3.12)	1.12 (0.76–1.66)	1.52 (1.15–2.01)
		Wald *χ*^2^ = 12.85, ***p* < 0.001**	Wald *χ*^2^ = 0.34 *p* = 0.558	Wald *χ*^2^ = 8.75, ***p* = 0.003**
WHO		*n* = 115	*n* = 123	*n* = 238
	0	1	1	1
	1	1.62 (1.07–2.47)	1.46 (0.95–2.24)	1.54 (1.14–2.08)
	2	0.97 (0.43–2.21)	1.22 (0.63–2.37)	1.09 (0.64–1.85)
		Wald *χ*^2^ = 5.79, *p* = 0.055	Wald *χ*^2^ = 3.01, *p* = 0.222	Wald *χ*^2^ = 8.55, ***p* = 0.014**
Lymph node status		*n* = 115	*n* = 123	*n* = 238
	Negative	1	1	1
	Positive	3.15 (1.76–5.62)	1.61 (0.95–2.74)	2.24 (1.50–3.33)
		Wald *χ*^2^ = 15.03, ***p* < 0.001**	Wald *χ*^2^ = 3.08, *p* = 0.079	Wald *χ*^2^ = 15.82, ***p* < 0.001**
Tumour stage		*n* = 114	*n* = 122	*n* = 236
	1/2	1	1	1
	3/4	1.92 (1.19–3.11)	1.47 (0.97–2.23)	1.67 (1.22–2.28)
		Wald *χ*^2^ = 7.16, ***p* = 0.008**	Wald *χ*^2^ = 3.34, *p* = 0.068	Wald *χ*^2^ = 10.13, ***p* = 0.002**
Tumour grade		*n* = 112	*n* = 120	*n* = 232
	Well	1	1	1
	Moderate	0.58 (0.36–0.94)	0.95 (0.42–2.12)	0.77 (0.47–1.28)
	Poor	0.75 (0.39–1.43)	1.25 (0.53–2.94)	1.02 (0.58–1.80)
		Wald *χ*^2^ = 5.17, *p* = 0.075	Wald *χ*^2^ = 1.56, *p* = 0.460	Wald *χ*^2^ = 3.15 *p* = 0.207
Local invasion		*n* = 115	*n* = 122	*n* = 237
	No	1	1	1
	Yes	1.30 (0.86–1.97)	1.24 (0.85–1.81)	1.27 (0.96–1.68)
		Wald *χ*^2^ = 1.56, *p* = 0.211	Wald *χ*^2^ = 1.20, *p* = 0.273	Wald *χ*^2^ = 5.06 ***p* = 0.025**
Maximum tumour diameter		*n* = 111	*n* = 118	*n* = 229
	**<30 **mm	1	1	1
	**≥30 **mm	1.28 (0.84–1.95)	1.36 (0.91–2.03)	1.33 (1.00–1.77)
		Wald *χ*^2^ = 1.36, *p* = 0.244	Wald *χ*^2^ = 2.25, *p* = 0.134	Wald *χ*^2^ = 3.78 *p* = 0.052
Diabetes mellitus		*n* = 112	*n* = 121	*n* = 233
	No	1	1	1
	Yes	0.96 (0.54–1.69)	0.90 (0.55–1.49)	0.92 (0.64–1.33)
		Wald *χ*^2^ = 0.02, *p* = 0.875	Wald *χ*^2^ = 0.20, *p* = 0.653	Wald *χ*^2^ = 0.18, *p* = 0.673
Gender		*n* = 115	*n* = 123	*n* = 238
	Male	1	1	1
	Female	1.19 (0.78–1.81)	1.20 (0.80–1.81)	1.19 (0.89–1.60)
		Wald *χ*^2^ = 0.66, *p* = 0.418	Wald *χ*^2^ = 0.76, *p* = 0.383	Wald *χ*^2^ = 1.40, *p* = 0.237
Age, years		*n* = 115	*n* = 123	*n* = 238
	≥64	1	1	1
	<64	1.33 (0.87–2.02)	0.89 (0.60–1.33)	1.07 (0.81–1.42)
		Wald *χ*^2^ = 1.74, *p* = 0.188	Wald *χ*^2^ = 0.32, *p* = 0.570	Wald *χ*^2^ = 0.23, *p* = 0.634
Smoking		*n* = 108	*n* = 113	*n* = 221
	Never smoker	1	1	1
	Ex-smoker	0.91 (0.57–1.46)	1.28 (0.82–1.98)	1.08 (0.79–1.49)
	Current smoker	0.92 (0.52–1.62)	1.48 (0.77–2.85)	1.13 (0.74–1.73)
		Wald *χ*^2^ = 0.17, *p* = 0.920	Wald *χ*^2^ = 1.94, *p* = 0.380	Wald *χ*^2^ = 0.42, *p* = 0.810
DPD expression		*n* = 115	*n* = 123	*n* = 238
	Low	1	1	1
	High	2.07 (1.22–3.53)	1.47 (0.91–2.37)	1.73 (1.21–2.49)
		Wald *χ*^2^ = 7.22, ***p* = 0.007**	Wald *χ*^2^ = 2.43, *p* = 0.119	Wald *χ*^2^ = 8.86, ***p* = 0.003**
hENT1 expression		*n* = 113	*n* = 118	*n* = 231
	Low	1	1	1
	High	1.19 (0.80–1.78)	0.56 (0.38–0.82)	0.84 (0.63–1.12)
		Wald *χ*^2^ = 0.74, *p* = 0.390	Wald *χ*^2^ = 8.98, ***p* = 0.003**	Wald *χ*^2^ = 1.44, *p* = 0.230

Data in bold indicate significant relationships.

### Multivariate analyses of prognostic factors in the respective treatment arms

A multivariate Cox regression model for prognosis showed that treatment arm was not significant (*p* = 0.138), whilst DPD expression was (*p* = 0.003), and hENT1 expression was not significant (*p* = 0.327). The interaction of treatment arm and DPD expression was not significant (*p* = 0.303), but the interaction of treatment arm and hENT1 expression was (*p* = 0.009).

Furthermore, multivariate analysis revealed that DPD expression status, along resection margin status, WHO performance status, and lymph node involvement were independent prognostic factors in the 5FU/FA treated subgroup but not the gemcitabine-treated group (Table 2). High DPD expression was significantly associated with survival in the 5-FU/FA treated group (HR 3.30; 95% CI 1.89–5.77; *p* < 0.001) but not in the gemcitabine-treated group (HR 1.62; 95% CI 0.97–2.69; *p* = 0.065).

**Table 2:** Multivariate analyses for survival of clinical and pathological risk factors and DPD tumour expression in 5-fluorouracil with folinic acid and gemcitabine-treated arms

Multivariate Analysis							
Variable		5-fluorouracil /folinic acid (*n* = 115)			Gemcitabine (*n* = 123)		
		HR (95% CI)	Wald *χ*^2^	*p*	HR (95% CI)	Wald *χ*^2^	*p*
Country			6.55	0.050	Not included		
Resection margin	Negative	1	7.75	0.005	1	0.30	0.585
	Positive	1.95 (1.22–3.11)			1.12 (0.75–1.67)		
WHO	0	1	8.47	0.013	1	3.38	0.184
	1	2.15 (1.28–3.60)			1.47 (0.95–2.27)		
	2	1.72 (0.76–3.89)			1.06 (0.53–2.13)		
Lymph node status	Negative	1	8.94	0.003	1	3.76	0.053
	Positive	2.88 (1.44–5.77)			1.71 (0.99–2.95)		
DPD expression	0/1	1	17.71	<0.001	1	3.41	0.065
	2/3	3.30 (1.89–5.77)			1.62 (0.97–2.69)		

### Integrating DPD and hENT1 as predictive biomarkers for adjuvant chemotherapy

**Fig. 1:**
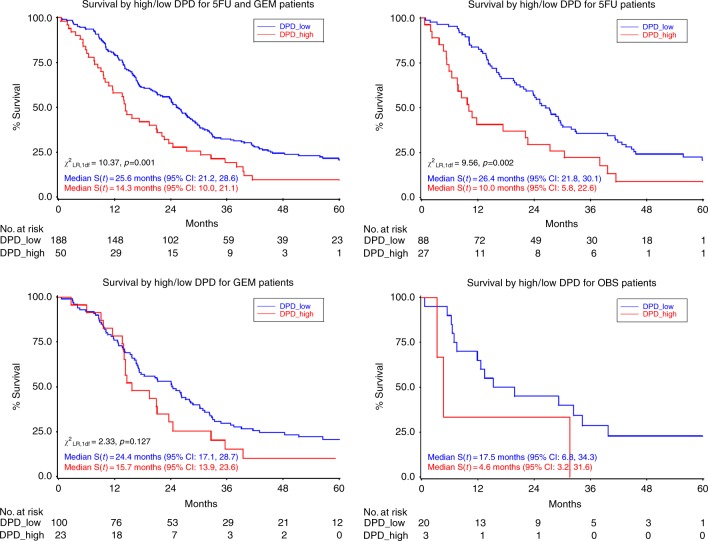
Kaplan–Meier survival curves and median overall survival for DPD-low vs. DPD-high tumour expression in the entire chemotherapy-treated population (5FU/FA plus gemcitabine), 5FU/FA treated patients, gemcitabine-treated patients, and the small observational (OBS) population

In the combined chemotherapy-treated group (5FU/FA and gemcitabine), the median (95% CI) overall survival time was 25.6 (21.2–28.6) months in patients with low DPD tumour expression and 14.3 (10.0–21.1) months in those with high DPD expression (*χ*^2^_LR,1 df = _10.4, *p* = 0.001, Fig. 1). This difference remained statistically significant in the 5-FU/FA arm treated subgroup, where median (95% CI) overall survival was 26.4 (21.8–30.1) months with low DPD tumour expression and 10.0 (5.8–22.6) months in those with high DPD expression (*χ*^2^_LR, 1 df_ = 9.56, *p* = 0.002). Overall median (95% CI) survival in gemcitabine-treated patients was not significantly different according to DPD status (24.4 (17.1–28.7) months in those with low DPD tumour expression and 15.7 (13.9–23.6) months in those with high DPD expression (*χ*^2^_LR, 1 df_ = 2.33, *p* = 0.127). The small population of patients randomised to observation was separately analysed. Patients with low DPD expression (*n* = 20) had an overall median (95% CI) of 17.5 (6.8–34.3) months compared to 4.6 (3.2–31.6) months in those with high DPD *(n* = 3) expression (Fig. 1). Due to the low numbers in this subset of patients, no *p* values were calculated and further statistical calculations or subdivisions were not performed.

Patients with high and low hENT1 tumour expression were subdivided according to high and low DPD tumour expression (Table 3 and Supplementary Figure 5). As we have previously reported, high hENT1 expression was associated with more favourable survival in gemcitabine-treated patients.^14^ We found no evidence for an additional prognostic value of DPD when added to hENT1 status in gemcitabine-treated patients. The median (95% CI) overall survival of patients treated with gemcitabine with high hENT1 intra-tumoural expression and also with low intra-tumoural DPD expression was 26.3 (17.2–33.0) months compared to 22.3 (9.6–39.5) months in those patients instead with high DPD expression, which was not significantly different (*p* = 0.360). The median (95% CI) overall survival of patients treated with gemcitabine with low hENT1 intra-tumoural expression and also low DPD intra-tumoural expression was 18.0 (7.6–15.3) months and 14.0 (9.1–15.7) months for patients with low hENT1 and high DPD intra-tumoural expression (*p* = 1.000).

**Table 3:** Median overall survival durations in subgroups based on combined hENT1 and DPD tumour expression status

Treatment arm	Subgroup	Number of patients	Median overall survival (95% CI) months	*P* value (raw)	*P* value post Bonferroni correction
5-fluorouracil with folinic acid	hENT1-high DPD-high	9	17.3 (0.6–38.0)	0.81	1.000
	hENT1-high DPD-low	39	26.0 (19.8–30.1)		
	hENT1-low DPD-high	17	9.7 (5.3–30.4)	**0.002**	**0.014**
	hENT1-low DPD-low	47	29.2 (19.5–41.9)		
Gemcitabine	hENT1-high DPD-high	12	22.3 (9.6–39.5)	0.060	0.360
	hENT1-high DPD-low	57	26.3 (17.2–33.0)		
	hENT1-low DPD-high	11	14.0 (9.1–15.7)	0.730	1.000
	hENT1-low DPD-low	38	18.0 (7.6–15.3)		

Data in bold indicate significant relationships.

Similarly, in patients with high hENT1 intra-tumoural expression treated with 5FU/FA, there was no significant difference between those who also had high or low DPD intra-tumoural expression with a median (95% CI) overall survival of 17.3 (0.6–38) and 26.0 (19.8–30.1) months respectively, (*p* = 1.000). However, in patients with low hENT1 expression treated with 5FU/FA, intra-tumoural DPD expression added significant predictive value. Thus, patients with low hENT1 and low DPD tumour expression treated with 5FU/FA had a median (95% CI) overall survival of 29.2 (19.5–41.9) months compared to 9.7 (5.3–30.4) months in those with low hENT1 and high DPD tumour expression (*χ*^2^_LR_ = 9.28, *p*[raw] = 0.002, *p*[post Bonferroni correction] = 0.014).

## DISCUSSION

In the present study, intra-tumoural DPD expression status was analysed in the ESPAC-3(v2) population of patients with pancreatic adenocarcinoma randomised to postoperative chemotherapy with 5FU/FA or gemcitabine. Given the key role of DPD in the catabolism of 5FU, we hypothesised that low intra-tumoural expression of DPD would result specifically in increased overall survival in patients treated with 5FU/FU. We found that DPD tumour expression was associated with reduced overall survival. Intra-tumoural DPD expression was also significant in the 5FU/FA arm but not in the gemcitabine arm. As previously shown high hENT1 tumour expression was associated with increased survival in patients treated with gemcitabine but not in those treated with 5FU/FA.

Given the previously reported predictive value of hENT1 tumour expression for adjuvant gemcitabine, we also explored the potentially additional value of DPD tumour expression in those high or low hENT1 intra-tumoural expression subgroups. In patients with high hENT1 tumour expression treated with gemcitabine, either low or high DPD expression showed a favourable median overall survival. Similarly, in 5-FU/FA treated patients with high hENT1 tumour expression no significant difference between high or low DPD tumour expression was observed. This suggests that if hENT1 tumour expression is high, evaluation of DPD tumour expression will not add any useful information, and these patients should generally be recommended for gemcitabine given a more tolerable toxicity profile. Another option, in situations where gemcitabine is unsuitable, would be a 5FU/FA bolus regimen other than the Mayo Clinic schedule or infusion regimen.

In patients with low hENT1 tumour expression treated with gemcitabine, survival was poor irrespective of DPD tumour expression. These data confirm that hENT1 tumour expression is a potentially useful predictive biomarker for improved survival with adjuvant gemcitabine. However, for patients with low hENT1 tumour expression treated with 5FU/FA, evaluation of DPD tumour expression provided additional predictive value. Patients with low DPD tumour expression treated with 5FU/FA survived significantly longer than patients with high DPD tumour expression. This suggests that there is a subgroup of patients with low hENT1 tumour expression and with low DPD tumour expression that derive significant survival benefit from adjuvant 5FU/FA. Conversely, the subgroup of patients with hENT1-low tumour expression and with high DPD tumour expression has a poor survival outcome whether treated with 5FU/FA or gemcitabine. We hypothesise that the additional prognostic information from intra-tumoural DPD expression status could be integrated with the hENT1 expression status to guide the selection of adjuvant chemotherapy regimen. We can conclude the following.

1. Patients with high hENT1 tumour expression are likely to derive a survival benefit from gemcitabine therapy irrespective of DPD tumour expression status. Analysis of DPD expression will not add any useful information.
2. In patients with low hENT1 tumour expression status, gemcitabine is less efficacious. For these patients DPD tumour expression may be analysed for additional prognostic information.
  - Patients with low hENT1 and low DPD tumour expression have a favourable prognosis with 5FU/FA treatment (median overall survival = 29.2 months).
  - Patients with high hENT1 and high DPD tumour expression have a poor prognosis whether given 5FU/FA or gemcitabine (9.7 and 14 months median overall survival, respectively). In these patients novel agents or combination regimens may be needed to improve survival.

Earlier studies investigating DPD tumour expression in pancreatic cancer were performed in smaller and/or non-controlled patient populations of Asian origin and involved the use of S-1 and/or combination with gemcitabine or radiotherapy.^11,17–23^ Asian individuals handle the metabolism of fluoropyrimidines differently from Europeans in part due to genotypic differences such as in CYP2A6 (which converts tegafur in S-1 to 5FU.^29^) The present study provides novel evidence as it was performed in a randomised controlled setting in patients who were primarily of European origin, and notably receiving single agent regimens.

Planned biomarker analyses of the ESPAC-4 population^8^ will assess whether hENT1, DPD and/or other tumour expression biomarker candidates are suitable for the identification of patients particularly benefitting from the gemcitabine plus capecitabine combination regimen. It is plausible that patients with low hENT1 and high DPD tumour expression may be resistant to gemcitabine and 5FU/FA individually and to the gemcitabine/capecitabine combination requiring alternative adjuvant strategies. If this is confirmed by biomarker analysis of the ESPAC4 trial biospecimens, prospective trials of therapies acting independently of hENT1 and DPD would be warranted in this population.

In conclusion, intra-tumoural DPD expression was a negative prognostic biomarker for patients with pancreatic adenocarcinoma undergoing postoperative chemotherapy. Intra-tumoural hENT1 expression was confirmed to be a predictive marker for gemcitabine treatment, and the additional prognostic value of DPD tumour expression may be used to estimate the survival in patients with low hENT1 tumour expression, where low DPD tumour expression indicates better prognosis at least for patients treated with 5FU/FA. Patients with low hENT1 and high DPD tumour expression present a particular challenge, and novel agents and/or combination regimens will be needed to improve survival for this subgroup.

## Electronic supplementary material

Supplementary Online Data
