# Supplementary material for: Expression of dihydropyrimidine dehydrogenase (DPD) and hENT1 predicts survival in pancreatic cancer
Source: Br J Cancer. 2018 Mar 8;118(7):947–54. doi: 10.1038/s41416-018-0004-2 (PMC5931115; doi:10.1038/s41416-018-0004-2)
Supplement: Supplementary file 1 — Supplementary Online Data [file 41416_2018_4_MOESM1_ESM.docx]

**SUPPLEMENTARY ONLINE DATA**

**SUPPLEMENTARY MATERIALS AND METHODS**

**Summary: Validation and quality assessment of the primary anti-DPD antibody**

The primary antibody (rabbit-anti-DPD, Abcam *Ab 134922*, Abcam, Cambridge, UK) was validated in accordance with ESPAC-T steering committee policy. The quality assessment included: (1) the detection of a band of presumed size on Western blot with lysates from five different pancreatic cancer cell lines (Supplementary Figure 1); (2) siRNA-mediated knockdown of the DPD and the attenuation/disappearance of this band on Western blot (Supplementary Figure 2); and (3) the clear attenuation/disappearance of the staining identified in formalin fixed and paraffin embedded cell pellets of the respective knockdown cell lines (Supplementary Figure 3).

**Western Blot Analysis**

Pancreatic cell lines (BxPC-3, CFPAC, MIA PaCa-2, PANC-1 and SUIT-2) were cultured at 37°C with 5.0% CO_2_ and harvested when 80-90% confluent. Cell pellets were lysed in RIPA lysis buffer (50 mM Tris-HCl pH 8.0, 150 mM NaCl, 1% Igepal CA-630, 0.5% deoxycholate, 0.1% sodium dodechylsulphate [SDS]) and separated by SDS-PAGE according to molecular weight. Samples were loaded at equal concentrations of 20 µg protein/lane. Following transfer of proteins onto a polyvinylidene (PVDF) membrane non-specific proteins were blocked by incubation of the membrane in 5% non-fat milk (Biorad Labs., Hertfordshire, UK) at 4^o^C overnight before being probed with a rabbit monoclonal anti-DPD antibody (Abcam *ab134922*) at a dilution of 1:1000 for 1 hour at room temperature. Membranes were then washed repeatedly in T-PBS (phosphate-buffered saline with 0.1% Tween-20) for 1 hour followed by incubation with a secondary HRP-conjugated anti-rabbit antibody (Dako) at a dilution of 1:1000 for 1 hour. Subsequent washing in T-PBS was done and the membrane then prepared for chemiluminescence analysis. Membranes were then stripped and reprobed for β-actin to ensure equal protein loading of samples.

Supplementary Figure 1A shows hybridisation of the Abcam antibody to proteins in five different cell lysates. DPD is reported to have a molecular weight of 110 kDa which is consistent with the single band here detected.

**siRNA mediated knock down of DPD in SUIT-2 pancreatic cancer cells**

DPD siRNA knockdown was investigated using SUIT-2 cells and a commercially available pool of siRNA strands targeting DPD mRNA (Dharmacon, GE Healthcare Ltd., Little Chalfont, United Kingdom). SUIT-2 cells were transfected with Lipofectamine 2000® (Life Technologies Ltd., Paisley, United Kingdom) and the respective siRNA pool to a final siRNA concentration of 20 nM. Three different conditions were used as negative control transfections: ‘off-target’ siRNA pool transfected cells [OT], RISC-free siRNA transfected cells [RF], and ‘wild type’ [WT] cells where the siRNA was omitted, respectively. The standard OT and RF siRNA pools were designed and provided by Dharmacon.

Following 72 hours incubation in 37°C in 5% CO_2_, cells were split with Trypsin-EDTA (Life Technologies) for five minutes in 37°C. Following a PBS wash cells were centrifuged and divided into two aliquots, of which one was immediately put in formalin for subsequent paraffin embedding, and the other aliquot immediately put in RIPA lysis buffer for subsequent SDS-PAGE electrophoresis and Western blot analysis. The supposed DPD 110 kD band was hardly detectable in the lysate of the DPD knock down treated cells. On the other hand WT, OT, and RF cells all displayed a distinct band of the proper size (Supplementary Figure 2).

**Immunocytochemistry of SUIT-2 cells**

SUIT-2 cell aliquots underwent formalin fixation and agar embedding according to the routines of our laboratory, and were subsequently dehydrated and embedded in paraffin blocks which were cut in 5 µm sections for the following immunocytochemical staining. The sections underwent rehydration and antigen retrieval with the PT-LINK® pH 9.0 buffer system (Dako, Glostrup, Denmark) in 95°C, according to the supplier’s recommendations. Sections then underwent incubation with peroxidase blocker (Dako) for 10 minutes and subsequent TBST (Tris-buffered saline with 0.05% Tween-20) washes followed by the incubation with the anti-DPD antibody at a dilution of 1:2000 for 1 hour in room temperature. Following repeated TBST washes sections were incubated with HRP-conjugated anti-rabbit-antiserum (Dako) and diaminobenzidine (DAB) staining according to supplier’s recommendations. Sections were counterstained with hematoxylin and stepwise dehydrated before mounting. SUIT-2 cells treated with the knock down pool were collected 72-hours post-transfection and displayed in general very weak or absent staining (Supplementary Figure 3A). On the other hand cells treated with control pools displayed a clear cytoplasmic staining with intensity varying among the individual cells (Supplementary Figures 3B-C). There was little or no membranous or nuclear staining observed.

**SUPPLEMENTARY FIGURES**

**Supplementary Figure 1.** A) Western blot showing the supposed 110 kDa DPD band in five different cell lines. B) The membrane was stripped and reprobed with anti-β-actin-antibody and HRP-conjugated secondary antibody to ensure equal loading. 1) BxPC-3; 2) CFPAC; 3) MIA PaCa; 4) PANC-1; 5) SUIT-2.


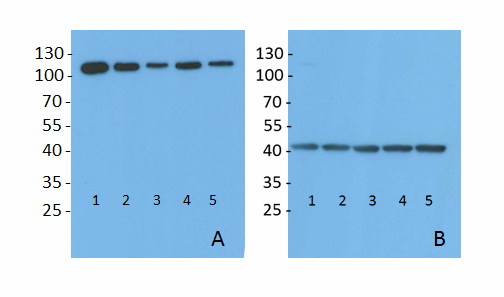


**Supplementary Figure 2.** A) Western blot depicting the disappearance of the 110 kD DPD band following transfection of an anti-DPD siRNApool into SUIT-2 cells. B) The membrane following stripping and reprobing with anti-β-actin antibody. MW: Molecular weight. WT: no siRNA. OT: Off target siRNA pool. RF: RISC-free siRNA pool. KD: Anti-DPD siRNA pool.


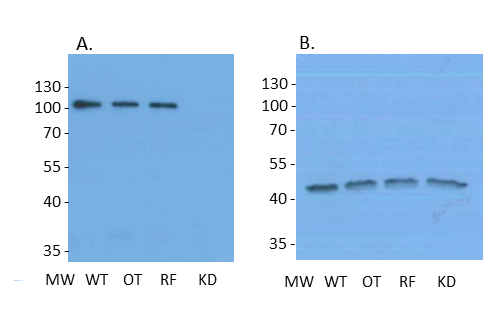


**Supplementary Figure 3.** A) Immunocytochemistry of anti-DPD siRNA treated SUIT-2 cells. B) ‘Off target’ siRNA control. C) RISC-free siRNA control. x200 magnification.

*
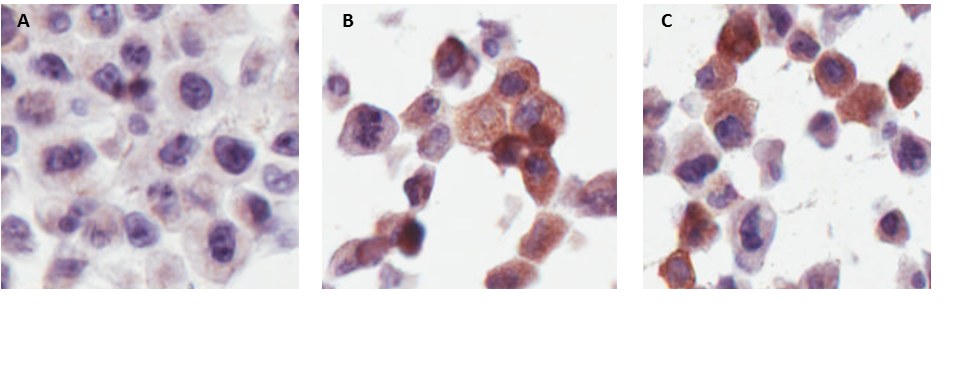
*

**Supplementary Figure 4.** Representative images of DPD immunohistochemical staining. A = DPD-0 (negative, *n*=94). B = DPD-1 (weak, *n*=114). C = DPD-2 (moderate, *n*=50). D = DPD-3 (strong, *n*=3). Arrow indicates a positively staining macrophage (working as internal positive control).


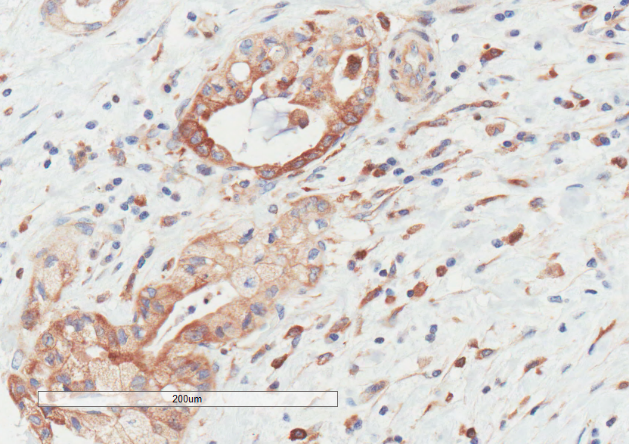

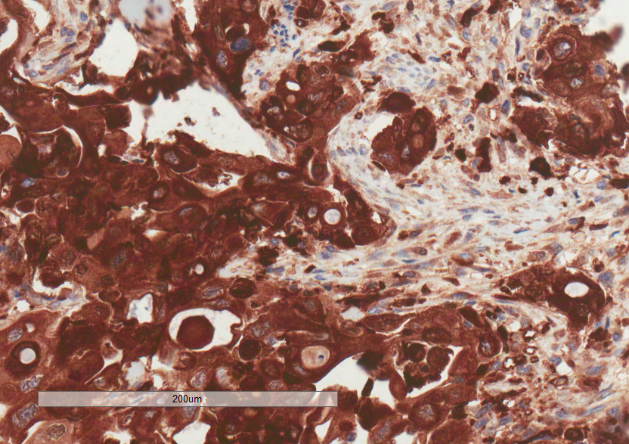

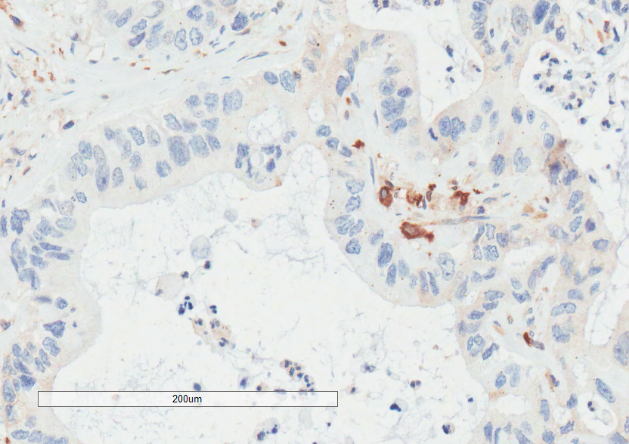

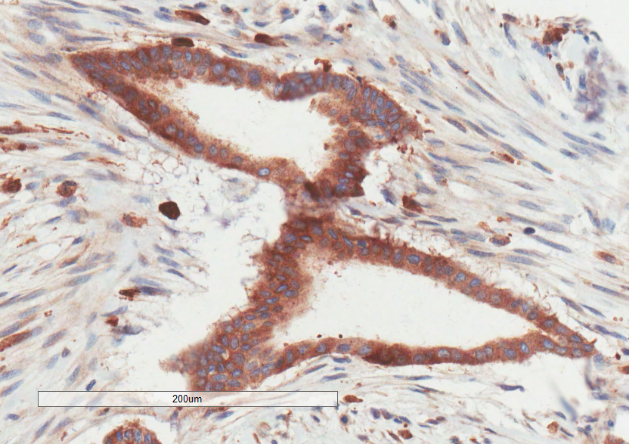


**A**

**B**

**C**

**D**

**Supplementary Figure 5.** Kaplan-Meier survival curves and median overall survival in the 5FU/FA and gemcitabine treated arms, for subgroups defined by the combination of hENT1 and DPD tumour expression status.

**
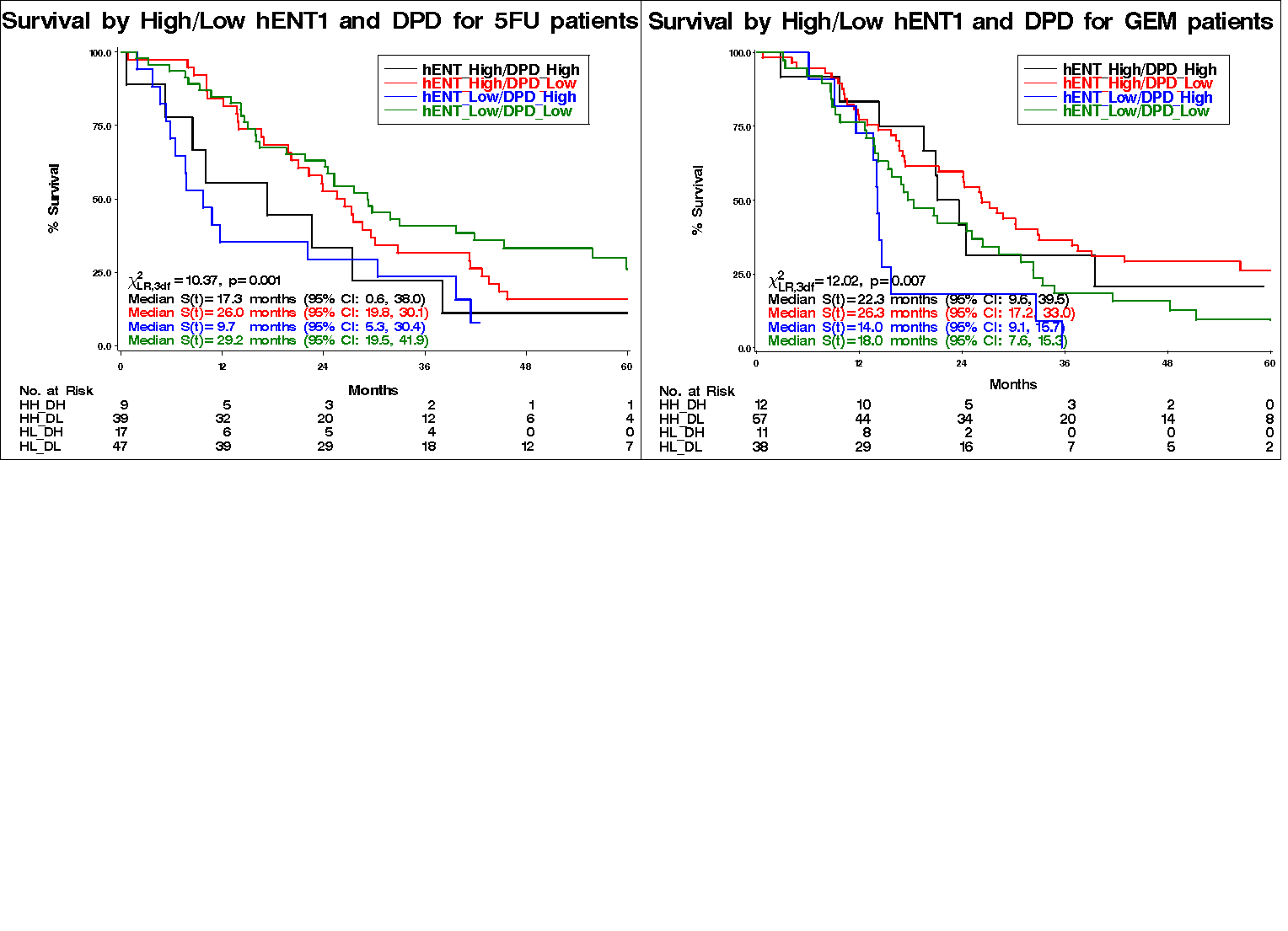
**

**SUPPLEMENTARY TABLES**

**Supplementary Table 1.** Demographic, surgery and pathology features of the 238 chemotherapy treated patients scored for DPD tumour expression.

| Demographics | | |  | | Total |
| --- | --- | --- | --- | --- | --- |
| Characteristic | | | **5-Fluorouracil/ folinic acid** | **Gemcitabine** |  |
|  | |  | ***n*=115** | ***n*=123** | ***n*=238** |
| Age Median (IQR) years | | | 62 (56-70) | 65 (57-70) | 64 (57-70) |
| Sex | **Female** | | 51 (44%) | 46 (37%) | 97 (41%) |
|  | **Male** | | 64 (56%) | 77 (63%) | 141 (59%) |
| WHO Performance Score | **0** | | 45 (39%) | 42 (34%) | 87 (37%) |
|  | **1** | | 57 (50%) | 67 (54%) | 124 (52%) |
|  | **2** | | 13 (11%) | 14 (11%) | 27 (11%) |
| Diabetes mellitus | **No** | | 89 (79%) | 92 (76%) | 181 (78%) |
|  | **NIDDM** | | 7 (6%) | 8 (7%) | 15 (6%) |
|  | **IDDM** | | 16 (14%) | 21 (17%) | 37 (16%) |
| Smoking status | **Never** | | 45 (42%) | 47 (42%) | 92 (42%) |
|  | **Past** | | 43 (40%) | 50 (44%) | 93 (42%) |
|  | **Present** | | 20 (19%) | 16 (14%) | 36 (16%) |
| Post-operative complications | **No** | | 90 (78%) | 91 (75%) | 181 (76%) |
|  | **Yes** | | 25 (22%) | 31 (25%) | 56 (24%) |
| Hospital stay | **Number** | | 110 | 113 | 223 |
| Median (IQR) days | | | 12 (10-17) | 13 (10-19) | 13 (10-18) |
| Post-Operative CA 19-9 | **Number** | | 89 | 87 | 176 |
| Median (IQR) KU/l | | | 30 (12-100) | 24 (11-54) | 28 (12-81) |
| Surgery to Randomization  Median (IQR) days | | | 49 (38-61) | 50 (39-59) | 49 (38-60) |

**Supplementary Table 2.** Relation between DPD tumour expression scores and clinical and pathological factors in the 238 chemotherapy treated patients.

| Characteristic | | Number | DPD mean score | | | | *p*-value |
| --- | --- | --- | --- | --- | --- | --- | --- |
|  |  |  | **0** | **1** | **2** | **3** |  |
| Resection Margin | **Negative** | 130 | 44 (34%) | 55 (42%) | 29 (22%) | 2 (2%) | 0.696 |
|  | **Positive** | 108 | 40 (37%) | 49 (45%) | 18 (17%) | 1 (1%) |  |
| Lymph Node Status | **Negative** | 44 | 11 (25%) | 26 (59%) | 7 (16%) | 0 (0%) | 0.160 |
|  | **Positive** | 194 | 73 (38%) | 78 (40%) | 40 (21%) | 3 (2%) |  |
| Tumour stage | **1** | 14 | 2 (14%) | 8 (57%) | 4 (29%) | 0 (0%) | 0.308 |
|  | **2** | 51 | 15 (29%) | 28 (55%) | 8 (16%) | 0 (0%) |  |
|  | **3** | 164 | 62 (38%) | 64 (39%) | 35 (21%) | 3 (2%) |  |
|  | **4** | 7 | 4 (57%) | 3 (43%) | 0 (0%) | 0 (0%) |  |
| Tumour grade | **Well** | 16 | 5 (31%) | 7 (44%) | 3 (19%) | 1 (6%) | 0.065 |
|  | **Moderate** | 151 | 58 (38%) | 68 (45%) | 25 (17%) | 0 (0%) |  |
|  | **Poor** | 65 | 18 (28%) | 27 (42%) | 18 (28%) | 2 (3%) |  |
| Local invasion | **No** | 128 | 44 (34%) | 53 (41%) | 30 (23%) | 1 (1%) | 0.431 |
|  | **Yes** | 109 | 39 (36%) | 51 (47%) | 17 (16%) | 2 (2%) |  |
| Resection Margin | **R0** | 127 | 44 (34%) | 55 (42%) | 29 (22%) | 2 (2%) | 0.696 |
|  | **R1** | 109 | 41 (38%) | 49 (45%) | 18 (17%) | 1 (1%) |  |
| Maximum Tumour Diameter | **<30mm** | 103 | 33 (32%) | 49 (48%) | 19 (18%) | 2 (2%) | 0.591 |
|  | **>30mm** | 126 | 49 (38%) | 51 (40%) | 25 (20%) | 1 (1%) |  |
| Diabetes mellitus | **No** | 182 | 69 (38%) | 76 (42%) | 34 (19%) | 2 (1%) | 0.408 |
|  | **Yes** | 51 | 14 (27%) | 26 (51%) | 11  (21%) | 1 (2%) |  |
| Gender | **Male** | 141 | 52 (37%) | 58 (41%) | 29 (21%) | 2 (1%) | 0.847 |
|  | **Female** | 97 | 32 (33%) | 46 (47%) | 18 (19%) | 1 (1%) |  |
| Age (Years) | **<65** | 117 | 39 (33%) | 53 (45%) | 23 (20%) | 2 (2%) | 0.870 |
|  | **>65** | 121 | 45 (37%) | 51 (42%) | 24 (20%) | 1 (1%) |  |
